# Supplementary material for: Ageing society and the challenge for social robotics: A systematic review of Socially Assistive Robotics for MCI patients
Source: PLoS One. 2023 Nov 30;18(11):e0293324. doi: 10.1371/journal.pone.0293324 (PMC10688856; doi:10.1371/journal.pone.0293324)
Supplement: S1 Fig — (DOCX) [file pone.0293324.s001.docx]

*Supporting figures.*

**S1 Fig.** This figure shows the percentage of studies in terms of risk of bias (Low risk in green; Some concerns in yellow; High risk in red) subdivided by RoB 2 scale macro-category.
