# Supplementary material for: Ageing society and the challenge for social robotics: A systematic review of Socially Assistive Robotics for MCI patients
Source: PLoS One. 2023 Nov 30;18(11):e0293324. doi: 10.1371/journal.pone.0293324 (PMC10688856; doi:10.1371/journal.pone.0293324)

**S2 Fig.** This figure illustrates the ratings of each macro category of RoB 2 and the final computation of risk of bias (“Overall”). The rating levels are divided into: Low risk (green); Some concern (yellow); High risk (red).


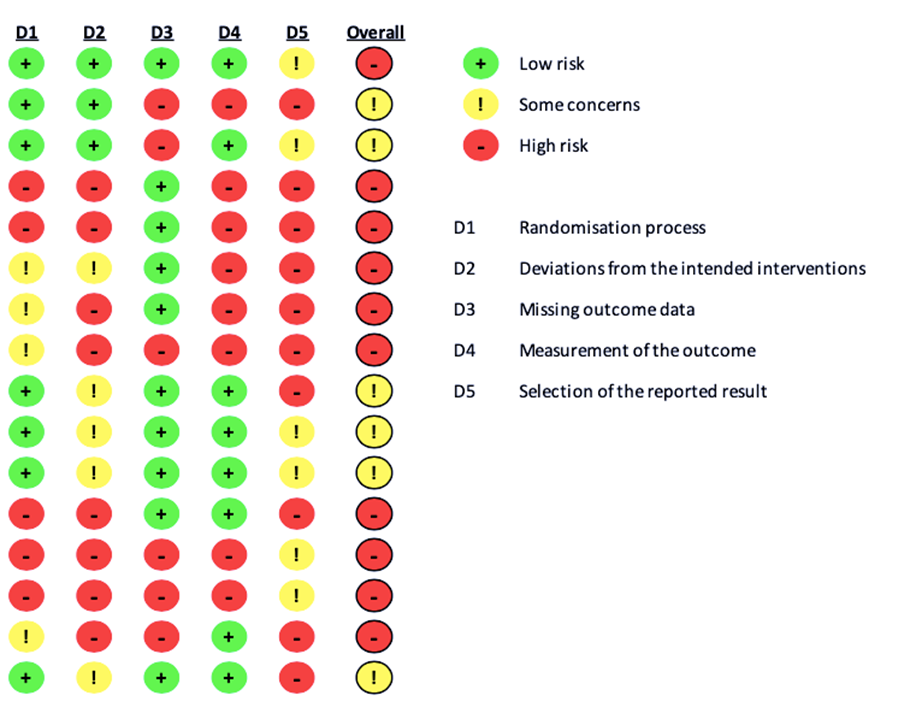

Supplement: S2 Fig — The rating levels are divided into: Low risk (green); Some concern (yellow); High risk (red). (DOCX) [file pone.0293324.s002.docx]
