# Supplementary material for: Ageing society and the challenge for social robotics: A systematic review of Socially Assistive Robotics for MCI patients
Source: PLoS One. 2023 Nov 30;18(11):e0293324. doi: 10.1371/journal.pone.0293324 (PMC10688856; doi:10.1371/journal.pone.0293324)
Supplement: S3 Fig — The rating levels are divided into: Yes + (green); Not applicable (yellow); No, or not enough information in article (red). (DOCX) [file pone.0293324.s003.docx]

**S3 Fig.** This figure illustrates the ratings of each study subdivided for every item of the GRACE scale. The rating levels are divided into: Yes + (green); Not applicable (yellow); No, or not enough information in article (red).
